# Synaptic plasticity and sensory-motor improvement following fibrin sealant dorsal root reimplantation and mononuclear cell therapy

**DOI:** 10.3389/fnana.2014.00096

**Published:** 2014-09-09

**Authors:** Suzana U. Benitez, Roberta Barbizan, Aline B. Spejo, Rui S. Ferreira, Benedito Barraviera, Alfredo M. Góes, Alexandre L. R. de Oliveira

**Affiliations:** ^1^Department of Structural and Functional Biology, Institute of Biology, University of CampinasCampinas, Brazil; ^2^Center for Studies of Venoms and Venomous Animals (CEVAP), University of Sao Paulo “Julio de Mesquita Filho,”Botucatu, Brazil; ^3^Department of Biochemistry and Immunology, Institute of Biological Sciences, Federal University of Minas GeraisBelo Horizonte, Brazil

**Keywords:** dorsal root rhizotomy, fibrin sealant, mononuclear cells, motor control, sensory recovery

## Abstract

Root lesions may affect both dorsal and ventral roots. However, due to the possibility of generating further inflammation and neuropathic pain, surgical procedures do not prioritize the repair of the afferent component. The loss of such sensorial input directly disturbs the spinal circuits thus affecting the functionality of the injuried limb. The present study evaluated the motor and sensory improvement following dorsal root reimplantation with fibrin sealant (FS) plus bone marrow mononuclear cells (MC) after dorsal rhizotomy. MC were used to enhance the repair process. We also analyzed changes in the glial response and synaptic circuits within the spinal cord. Female Lewis rats (6–8 weeks old) were divided in three groups: rhizotomy (RZ group), rhizotomy repaired with FS (RZ+FS group) and rhizotomy repaired with FS and MC (RZ+FS+MC group). The behavioral tests electronic von-Frey and Walking track test were carried out. For immunohistochemistry we used markers to detect different synapse profiles as well as glial reaction. The behavioral results showed a significant decrease in sensory and motor function after lesion. The reimplantation decreased glial reaction and improved synaptic plasticity of afferent inputs. Cell therapy further enhanced the rewiring process. In addition, both reimplanted groups presented twice as much motor control compared to the non-treated group. In conclusion, the reimplantation with FS and MC is efficient and may be considered an approach to improve sensory-motor recovery following dorsal rhizotomy.

## Introduction

Motor coordination is dependent on delicate sensory-motor integration, which is particularly evident in the spinal cord. In this sense, primary afferent inputs enter the dorsal horn and make synapses at different laminae, according to the nature of the information (Brodal and Rexed, 1953; Rexed, 1954). Many of these inputs project directly or indirectly to the motoneurons present in the ventral horn. Loss of sensorial information greatly affects motor behavior and constitutes an important medical problem (Rabert et al., 2004; Bigbee et al., 2007; Wu et al., 2012; Chew et al., 2013; Matsuura et al., 2013).

Spinal root injury has become a relatively common occurrence following vehicle accidents and is also related to complicated child-births (Carlstedt, 2009; Barbizan et al., 2013; Kachramanoglou et al., 2013; Spejo et al., 2013; Wu et al., 2013). It generally occurs in the brachial plexus, leading to loss of sensibility and paralysis of the limb ipsilateral to the injury. Importantly, such lesion may result in neuropathic pain, indicating that pathological circuitry rearrangements may take place (Carlstedt, 2009).

As mentioned, brachial plexus injuries may affect both motor and sensorial roots. In the last case, a series of molecular events affect the somatosensory pathways, resulting in extensive synaptic changes within the spinal cord (Scott, 2012). Such changes occur from weeks up to months post lesion. Also, sprouts from the dorsal roots are incapable of reaching deeper laminae of the spinal cord, since the central nervous system (CNS) environment is impeditive to axonal growth, mostly because of the astroglial extracellular matrix and due to the presence of myelin sheath components (Buchli and Schwab, 2005). The lack of neurotrophic factors (Lu et al., 2012) and the intense inflammatory reaction, combined with loss of blood vessels contribute to the development of an anti-regenerative environment. Nevertheless, the glial scar secreted mostly by astrocytes is important to stabilize the injured tissue and to accelerate the blood brain barrier reconstitution (Silver and Miller, 2004). Astrocyte activation is also important for the reuptake of glutamate, which might reduce glutamate excitotoxicity (Xanthos and Sandkuhler, 2014). The glial scar is however, mostly constituted of inhibiting components and blocks the regenerative process. Together with astrocytes, microglia becomes reactive after an injury and actively phagocyzes tissue debris, but also secrets inflammatory substances that act against axonal growth (De Leo et al., 2006). Therefore, it is important to balance the positive and negative effects of reactive glia.

Despite all changes in the spinal cord microenvironment, the current restorative surgical procedures are restricted only to reconnection of the ventral roots. Such procedure is focused on restoring some motor functions, discarding the sensory component. Due to this, a significant number of patients begin to obtain limited motor function recovery up to 1 year after surgery (Carlstedt, 2009). However, since the restoration of sensory functions does not occur (Carlstedt, 2009; Chew et al., 2013), a persistent imbalance of excitatory and inhibitory inputs remains and generates important changes in the CNS homeostasis.

Although the continuous pain is the more immediate problem, lesions in sensory pathways trigger significant changes in motor control (Scott, 2012), since the intraspinal circuits are directly affected, disturbing the motor coordination (Carlstedt and Havton, 2012; Carlstedt et al., 2012). Taking that into account, it is relevant to study lesions to dorsal roots alone and develop strategies that may allow the restoration of the motor-sensory integration. This may, in turn, significantly enhance the patient quality of life. Based on the facts above mentioned, the present work proposes the use of bone marrow mononuclear cells associated with fibrin sealant in order to reimplant dorsal roots and improve the necessary microenvironment for primary afferent recovery within the spinal cord. The fibrin sealant therapy resulted in a better synaptic circuitry recovery compared to non-treated group, thus resulting in significant improvement of gait and motor coordination. Moreover, cell therapy provided better recovery of glutamatergic circuits concomitant with sensory recovery.

## Methods

### Animals and experimental groups

Sixty-five adult female Lewis rats (LEW/HsdUnib) (6–8 weeks old) were obtained from the Multidisciplinary Center for Biological Investigation (CEMIB/UNICAMP) and housed under a 12-hour light/dark cycle with free access to food and water. The study was approved by the Institutional Committee for Ethics in Animal Experimentation (Committee for Ethics in Animal Use—Institute of Biology—CEUA/IB/UNICAMP, proc. n° 2357-1). All experiments were performed in accordance with the guidelines of the Brazilian College for Animal Experimentation. The animals were subjected to unilateral rhizotomy of the L4–L6 dorsal roots and divided into 3 groups: (1) RZ without reimplantation (RZ, *n* = 22); (2) RZ followed by root reimplantation with FS (RZ+FS, *n* = 23); and (3) RZ followed by root reimplantation with FS plus MC (RZ+FS+MC, *n* = 20). Detailed information about number of animals used per technique is provided in Table 1. Additional 5 animals (LEW-Tg EGFP F455/Rrrc) were used as mononuclear cells donors. The animals were killed 1 week (to reveal the removal of VGLUT1 afferent fibers and make sure the surgery was done correctly affecting various laminae within the spinal cord), 4 weeks (intermediate time to analyze synaptic rearrangement and start of functional recovery) and 8 weeks (late time in which is possible the consolidation of new synapses) post lesion. The contralateral side of the spinal cord and hind limb were used as the internal controls. For number of animals used in each experiment please check out tables of the Supplementary Material.

**Table 1:** **Number of animals allocated per group and technique**.

**Survival time**	**Technique**	**Groups**		
		**RZ**	**RZ+FS**	**RZ+FS+MC**
1 week	Immunohistochemistry/von-Frey	5	5	6
4 weeks	Immunohistochemistry/von-Frey/	6	6	5
	CatWalk	5	6	4
8 weeks	Immunohistochemistry	6	6	5
	Total	22	23	20

FS, fibrin sealant; MC, mononuclear cells; RZ, rhizotomy.

### Dorsal root rhizotomy

**Figure 1:**
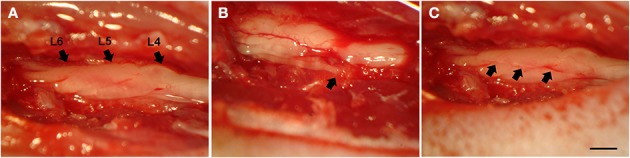
**Dorsal view of the lumbar spinal cord at L4-L6 level, following hemi-laminectomy. (A)** Lumbar intumescence showing intact L4–L6 dorsal roots. **(B)** Animal without reimplantation. The arrow shows one root disconnected from the spinal cord. **(C)** Animal subjected to reimplantation with fibrin sealant. The arrows (L6, L5, and L4, respectively) show the exact point of rhizotomy. Scale bar = 1 mm.

The rats were anesthetized with ketamin (50 mg/Kg, Fort Dodge, USA) and xylasine (10 mg/Kg, Köning, Argentina) and subjected to unilateral rhizotomy of the lumbar dorsal roots (Figure 1). Unilateral rhizotomy was performed at the L4–L6 lumbar dorsal roots after unilateral laminectomy (at the right side). A longitudinal incision was made to open the dural sac and the dorsal roots associated with the lumbar intumescence could be identified and cut 2 mm from the surface of the spinal cord with a micro scissor. After lesion, the roots were repositioned to their original position and the musculature and skin were sutured. Chlorhydrate of tramadol was administrated by gavage after the surgical procedures (20 mg/kg) and 2.5 mg/day (dissolved in drinking water) for 5 days. All surgical procedures were done following a previous work (Oliveira et al., 2003).

### Reimplantation of the sensory roots

In the RZ+FS and RZ+FS+MC groups, the roots were replaced on the dorsal surface of the lumbar spinal cord at the rhizotomy site (Figure 1C) with the aid of FS or FS plus MC (3 × 10^5^ cells in 3 μl of DMEM).

### Fibrin sealant

The fibrin sealant used in this study was provided by the Center for the Study of Venoms and Venomous Animals (CEVAP), UNESP, Brazil (patents BR 10 2014 011432 7 and BR 10 2014 011436 0) (Barros et al., 2009, 2011; Gasparotto et al., 2014). The sealant is composed of three separate solutions and homogenized at the time of use in a customized volume: (1) fibrinogen, (2) calcium chloride, and (3) thrombin-like fraction. This material was stored at −20°C prior to use at room temperature. During surgical repair of the lesioned roots, the first two components were applied and the cut roots were returned to their original sites. The third component was then added for polymerization, in a total of 8 μl of solution. In the RZ+FS+MC group, mononuclear cells were injected at the lesion site after the second component. The reimplantation stability was tested by gently pulling the roots from the surface of the spinal cord. If the reimplantation was not stable, more fibrin glue was added.

### Mononuclear cells extraction

MC were extracted from transgenic Lewis rats (LEW-Tg EGFP F455/Rrrc), with the EGFP (Enhanced green fluorescent protein) gene under Ubiquitin C promoter control. They were imported from the Missouri University (EUA) and provided by Prof. Dr. Alfredo Miranda Góes, Federal University of Minas Gerais—UFMG, Brazil.

The EGFP rats were killed with halothane (Tanohalo, Cristália Chemicals and Pharmaceuticals), and the femur and tibia were dissected out from the muscular and connective tissue and removed. The cell isolation was done following the separation of mononuclear cells methods described by Sigma–Aldrich n° 1119 protocol. The mononuclear cells were associated with fibrin sealant and applied on the spinal cord in the same day of the extraction.

### Mononuclear cells flow cytometry characterization

Mononuclear cells were characterized through flow cytometry. Approximately 1 × 10^7^ cells were incubated for 30 min at 4°C with the antibodies shown in Table 2. For the non-conjugated anti-serum, 30 min incubation with a secondary antibody was carried out (anti-sheep IGG, NL 011, R&D Systems, MN, USA). After washing with PBS (phosphate buffered saline), the cells were fixed with 1% paraformaldehyde and acquired using a fluorescence-activated cell sorter (BD FACSCalibur, BD Biosciences, San Jose, CA, USA). Approximately 1 × 10^5^ events were acquired, using the acquisition software CELLQuest (BD Biosciences, San Jose, CA, USA). The data were analyzed with Flow Jo 7.65.5 software.

**Table 2:** **Antibodies used for flow cytometry**.

**Antibody (anti-rat)**	**Manufacturer/Cat. N°**	**Conjugated with**
CD3	BD Pharmingen/22015b	PE
CD11b	BD Pharmingen/554982	FITC
CD34	R&D Systems/AF6518	–
CD45	BD Pharmingen/ 554877	FITC

### Specimen preparation

After the predetermined survival times, the animals were anaesthetized with an overdose of anaesthetic (mixture of xylasine and ketamine,) and the vascular system was transcardially perfused with phosphate buffer 0.1 M (pH 7.4) and then perfused with 3.7% formaldehyde in phosphate buffer (150 ml of fixative per animal). The rats were killed 1, 4, and 8 weeks after surgery and their lumbar spinal cords were dissected out and post fixed in the same fixative solution overnight at 4°C. They were then cryopreserved for 24 h in 20% sucrose buffered solution and embedded in Tissue–Tek (Miles Inc., USA) and frozen at −35°C. Spinal cord transverse sections (12 μm thick), from L4–L6 lumbar segments, were obtained and transferred to gelatin-coated slides and stored at −20°C until use.

### Immunohistochemistry

Transverse sections of spinal cord were incubated with the primary antibodies detailed in Table 3. The primary antibodies were diluted in a solution containing 1% bovine serum albumin (BSA) in phosphate buffer and Tween × 100 (PBST). All sections were incubated overnight at 4°C in a moist chamber. After washing with PBST, the sections were incubated according to the primary host antibody (CY-2 or CY-3, Jackson Immunoresearch, CA, USA; 1:500) for 45 min in a moist chamber at room temperature. The sections were then rinsed in PBST, mounted in a mixture of glycerol/PBS (3:1) and observed in a Nikon Eclipse TS100 inverted microscope (Nikon, Japan).

**Table 3:** **Primary antibodies used for immunohistochemistry**.

**Antibody**	**Manufacturer/Cat. N°**	**IGG-anti**	**Mono or polyclonal**
Vesicular Glutamate Transporter 1 (VGLUT1)	Synaptic Systems/135 303	Rabbit	Polyclonal
Synaptophysin	DakoCytomation/M0776	Mouse	Monoclonal
Glutamate Acid Decarboxylase 65 (GAD65)	Abcam/26113	Mouse	Monoclonal
Glial Fibrillary Acidic Protein (GFAP)	Abcam/7779	Rabbit	Polyclonal
Ionized calcium binding adaptor protein (Iba1)	Wako/019-19741	Rabbit	Polyclonal
Growth Associated Protein 43 (GAP-43)	Abcam/11136	Rabbit	Polyclonal

For quantitative measurements, three representative images of each region in the spinal cord from each animal were captured at a final magnification of ×200. A list of the Rexed laminae used for quantification for each antibody is represented in Table S1. Quantification was performed with IMAGEJ software (National Institute of Health, USA). The integrated density of pixels was systematically measured in specific laminae, based on the cytoarchitetonic distribution of afferents to the spinal cord. In this way, laminae I and II receive nociceptive inputs. Proprioceptive information reaches laminae IV–VI. Lamina IX is where the motoneurons are located and site of projection of afferents from different laminae (Rexed, 1954).

The integrated density of pixels was calculated for each section of spinal cord (ipsi and contralateral sides), and then a mean value for each spinal cord was obtained. It was calculated the Ipsilateral/Contralateral (IL/CL) ratio per animal and then established the average for each group. The data are represented as the mean ± standard error (SE). This technique is being used in previous publications from our group (Oliveira et al., 2004; De Freria et al., 2012; Victorio et al., 2012; Perez et al., 2013; Spejo et al., 2013).

### Functional analysis

Motor function was analyzed using the Peroneal functional index (PFI), Max contact max intensity, Print area, Regularity index, Stand and Max contact area parameters of CatWalk system (Noldus Inc., The Netherlands). In the CatWalk system, the animal crosses a walkway with an illuminated glass floor. A high speed video camera Gevicam (GP-3360, USA) equipped with a wide-angle lens (8.5 mm, Fujicon Corp., China) is positioned underneath the walkway and the paw prints are automatically recorded and classified by the software as the animal moves across the path. The paw prints from each animal were obtained before and after the surgery. Post-operative CatWalk data were collected twice a week for 8 weeks.

PFI was calculated as the distance between the third toe and hind limb pads (print length) and the distance between the first and fifth toes (print width). Measurements of these parameters were obtained from the right (lesioned) and left (unlesioned) paw prints, and the values were calculated using the following formula described by Bain et al. (1989):

$$\begin{array}{l} \text{PFI }=\;174.9\;\times \;((\text{EPL-NPL})\text{/NPL})\;+\;80.3\\ \;\times \;((\text{ETS }-\text{ NTS})\text{/NTS})\;-\;13.4 \end{array}$$

where N = normal, or non-operated side; E = experimental, or operated; PL = print length; TS = total toe spread, or distance between first to fifth toe.

The regularity index was expressed as an index of motor coordination. The others parameters used were expressed as a percentage of the IL/CL ratio.

Sensory function analysis was done using the electronic von-Frey (Vivancos et al., 2004), located at the Pain Neurobiology Laboratory, at Structural and Functional Department, UNICAMP. The nociceptive threshold was analyzed by an electronic analgesymeter. The plantar region of the rats was touched with a pipette tip adapted to the force transducer, which is connected with a digital counter that shows the force, in grams, used to trigger the paw flinch. The rats were kept inside acrylic boxes 20 min before the experiment for habituation. The hyperalgesia intensity was obtained before and after the surgery, and the results are shown as a mean ± SE of the measurements in each day for each paw of each animal. It was standardized that the strongest force applied to the footpad would be 90 g. If the animal did not respond up to this intensity of pressure, it was considered with total paw anesthesia. This experiment was conducted in a blinded manner.

### Statistical analysis

Parametric data were analyzed using Two-Way ANOVA, with Bonferroni *post hoc* test. The data are presented as the mean ± SE and the differences between groups were considered significant when the *P*-value was <0.05 (*), <0.01 (**), and <0.001 (***).

## Results

### Mononuclear cells

To test the homogeneity and to characterize the isolated MC, the expression of hematopoietic and non-hematopoietic markers were analyzed. The MC expressed low CD3 (1.52%) and a significant percentage of cells were positive to CD11b (13.2%), CD45 (92.2%), and CD34 (20.8%) (Figure S1). Thus, the isolated cells were considered as mononuclear cells.

Histological analysis of EGFP labeling in the injury site showed the MC up to 8 weeks post grafting, revealing the longtime of survival of such cells (see after). However, such cells did not penetrate into the spinal cord, remaining at the grafting site. Also, the cells maintained the typical spheroid morphology.

### Root reimplantation with FS and FS+MC improved motor function

**Figure 2:**
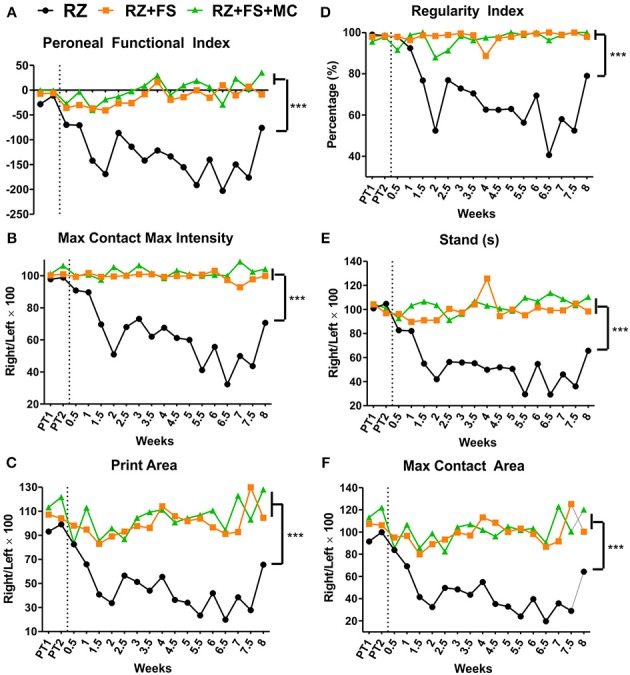
**Walking track test carried out using the Catwalk system (Noldus Inc., Holland). (A)** Peroneal functional index. **(B)** Max contact max intensity. **(C)** Print area. **(D)** Regularity index. **(E)** Stand (time in seconds that the animal stood over the paw ipsilateral to the lesion/repaired side). **(F)** Max contact area. The vertical dotted lines represent the moment of lesion/repair. CL, contralateral; FS, fibrin sealant; IL, ispsilateral; PT, pretest; MC, mononuclear cells; RZ, rhizotomy. ^***^p < 0.001.

The graphs of Figure 2, demonstrate that rhizotomy of dorsal roots leads to an extensive loss of motor coordination and motor function, which can be significantly improved by reimplantation and cell therapy. Such observation is valid for all parameters analyzed (Peroneal functional index, Max contact max intensity, Print area, Regularity index, Stand, Max contact area; *p* < 0.0001). Videos of the runs from the walking track test are available as supplementary material (Movies 1–3).

### Root reimplantation combined with MC therapy improved sensory function

**Figure 3:**
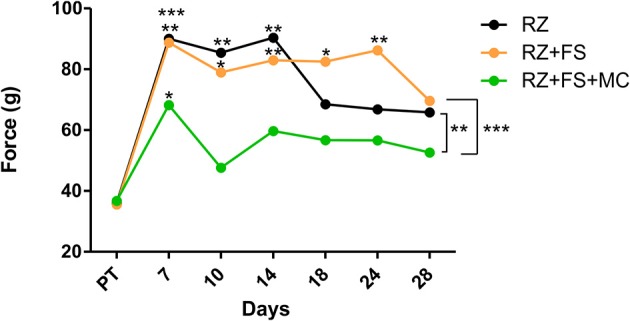
**Electronic von-Frey measurements (average values) obtained from the right hind paw (lesioned)**. The values are shown in grams (g) applied to trigger the “flinch” response. Statistical differences among groups are indicated with brackets. Asterisks above each dot represent the comparison between the time point and the respective pre-test. FS, fibrin sealant; PT, pretest; MC, mononuclear cells; RZ, rhizotomy.

The pain perception was measured by electronic von-Frey test. As shown in Figure 3, there was a difference among groups (*p* < 0.0001), so that RZ+FS+MC always showed the best performance. Seven days after lesion, it was observed that both RZ and RZ+FS groups presented total anesthesia of the ipsilateral hind paw. This situation remained unaltered up to 14 days in RZ group, and up to 24 days in RZ+FS group. On the other hand, 7 days after lesion, the RZ+FS+MC group already showed a flinch response compared to the other two groups, indicating partial recovery of proprioception. After 7 days, the measures of the same group did not differ from its pre-test values.

### The reimplantation with FS and FS+MC reduced astroglial reaction

**Figure 4:**
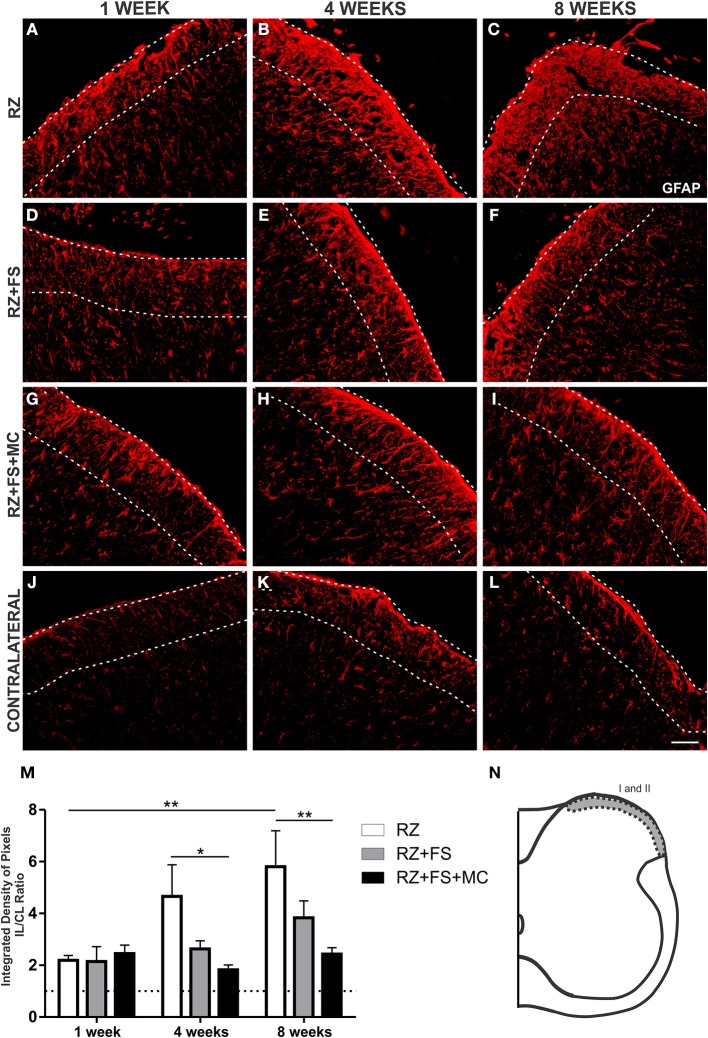
**Immunohistochemistry for glial fibrillary acidic protein (GFAP). (A–L)** Representative images of RZ, RZ+FS and RZ+FS+MC, 1, 4, and 8 weeks post lesion. **(M)** Quantification (ratio IL/CL) of the integrated density of pixels, obtained in the region delimited by dashed lines. **(N)** Representation of laminae I and II (total quantification area: 3.9 × 10^5^ μm^2^). CL, contralateral; FS, fibrin sealant; IL, ispsilateral; MC, mononuclear cells; RZ, rhizotomy. Scale bar = 50 μm. ^*^p < 0.05; ^**^p < 0.01.

The quantitative analysis in laminae I and II showed an increased expression of GFAP in the RZ group 8 weeks post lesion, compared to 1 week post lesion (*p* = 0.0124) (Figure 4). The RZ+FS+MC group prevented upregulation of GFAP compared to RZ group, 4 and 8 weeks post lesion (*p* < 0.05 and *p* < 0.01, respectively). The mean ± SE of the groups are shown in Table S2.

**Figure 5:**
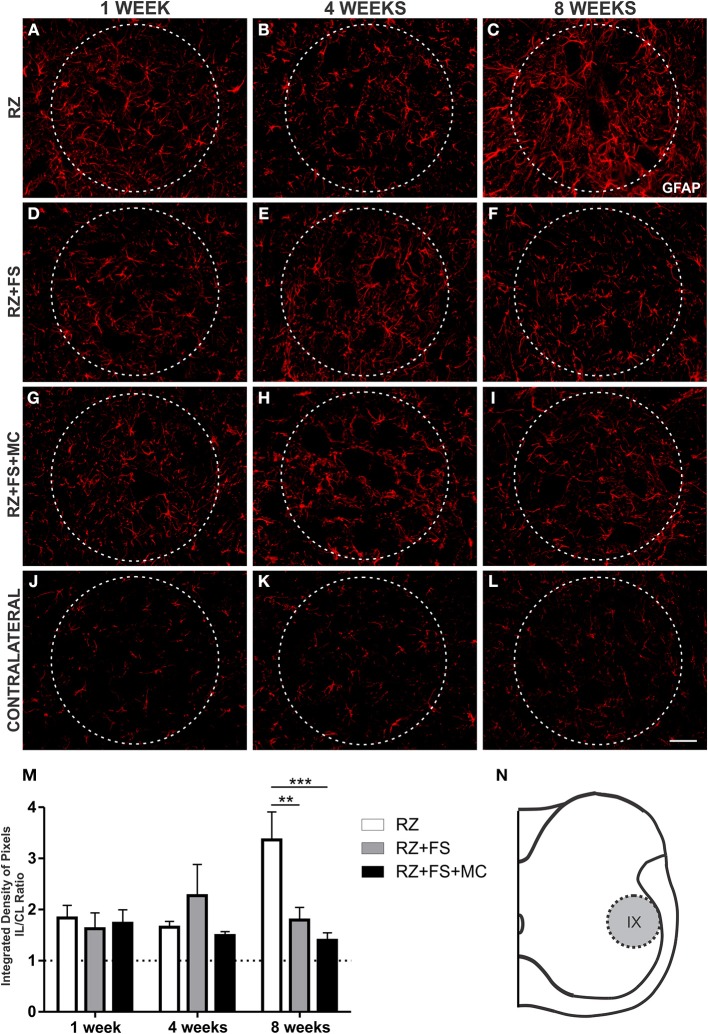
**Immunohistochemistry for glial fibrillary acidic protein (GFAP). (A–L)** Representative images of RZ, RZ+FS and RZ+FS+MC, 1, 4, and 8 weeks post lesion. **(M)** Quantification (ratio IL/CL) of the integrated density of pixels, obtained in the region delimited by dashed circles. **(N)** Representation of lamina IX (total quantification area: 8.5 × 10^5^ μm^2^). CL, contralateral; FS, fibrin sealant; IL, ispsilateral; MC, mononuclear cells; RZ, rhizotomy. Scale bar = 50 μm. ^**^p < 0.01; ^***^p < 0.001.

In lamina IX, GFAP quantification showed stronger upregulation in RZ, compared to RZ+FS (*p* < 0.01) and RZ+FS+MC groups (*p* < 0.001), 8 weeks post lesion (Figure 5). The mean ± SE of the groups are shown in Table S3.

### Root reimplantation and cell therapy reduce microglial reaction

**Figure 6:**
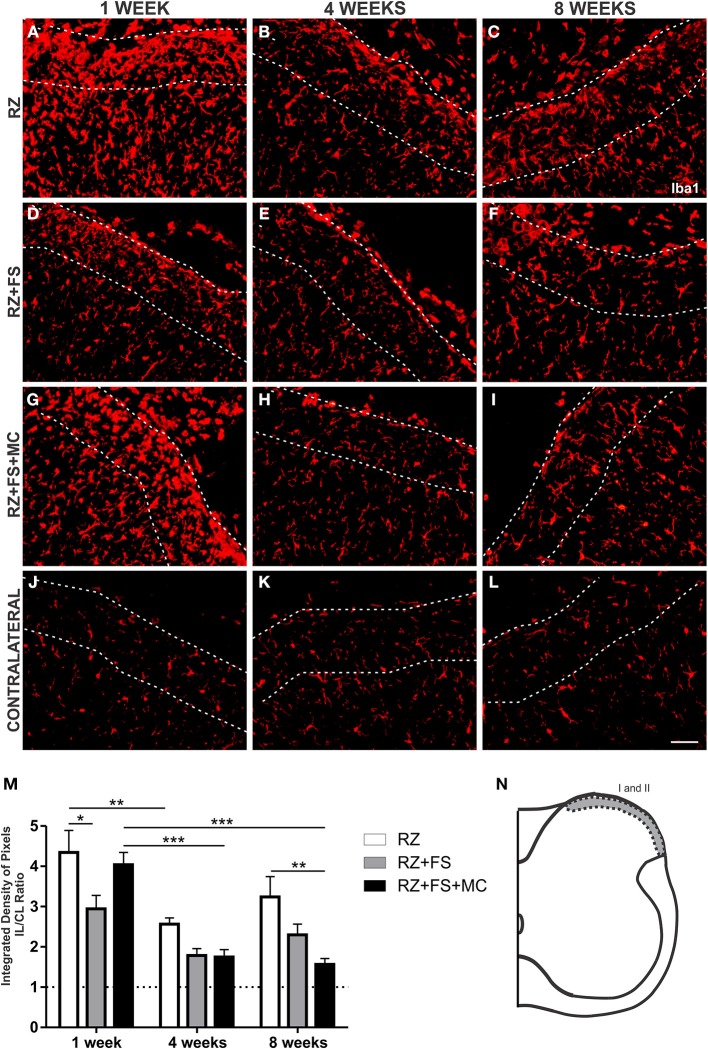
**Immunohistochemistry for ionized calcium binding adaptor protein (Iba1). (A–L)** Representative images of RZ, RZ+FS and RZ+FS+MC, 1, 4, and 8 weeks post lesion. **(M)** Quantification (ratio IL/CL) of the integrated density of pixels, obtained in the region delimited by dashed lines. **(N)** Representation of laminae I and II (total quantification area: 3.9 × 10^5^ μm^2^). CL, contralateral; FS, fibrin sealant; IL, ispsilateral; MC, mononuclear cells; RZ, rhizotomy. Scale bar = 50 μm. ^*^p < 0.05; ^**^p < 0.01; ^***^p < 0.001.

Dorsal root section led to upregulation of Iba1 in the superficial laminae of the spinal cord, 1 week post lesion. However, there was a natural decrease of the microglial reaction in the following weeks (*p* < 0.01) (Figure 6).

The reimplantation of the lesioned roots with FS alone resulted in acute decrease of Iba1 in laminae I and II that was statistically significant at 1 week post-surgery (*p* < 0.05).

Although the RZ+FS+MC group presented similar Iba1 levels as compared to the RZ alone, 1 week after rhizotomy, cell therapy led to a significant decrease of microglial reaction at later stages, namely 8 weeks after root reimplantation (*p* < 0.01). Interestingly, MC grafting prevented upregulation of Iba1 when comparing the first and eighth weeks post reimplantation (*p* < 0.001). The mean ± SE of the groups is shown in Table S4.

**Figure 7:**
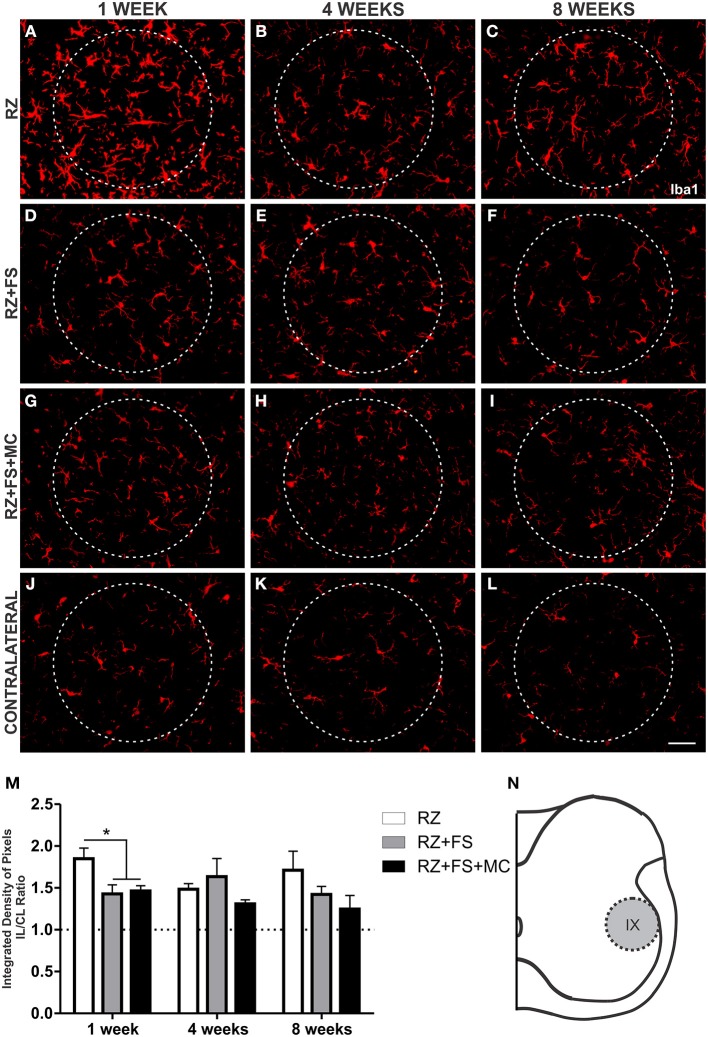
**Immunohistochemistry for ionized calcium binding adaptor protein (Iba1). (A–L)** Representative images of RZ, RZ+FS and RZ+FS+MC, 1, 4, and 8 weeks post lesion. **(M)** Quantification (ratio IL/CL) of the integrated density of pixels, obtained in the region delimited by dashed circles. **(N)** Representation of lamina IX (total quantification area: 8.5 × 10^5^ μm^2^). CL, contralateral; FS, fibrin sealant; IL, ispsilateral; MC, mononuclear cells; RZ, rhizotomy. Scale bar = 50 μm. ^*^p < 0.05.

Regarding Iba1 immunostaining at lamina IX, in the surroundings of motoneurons, a decreased expression of Iba1 could be depicted in RZ+FS and RZ+FS+MC compared to RZ, 1 week post lesion (*p* < 0.05) (Figure 7). The mean ± SE of the groups is shown in Table S5.

### FS and FS+MC increased VGLUT1 synaptic plasticity within the spinal cord

**Figure 8:**
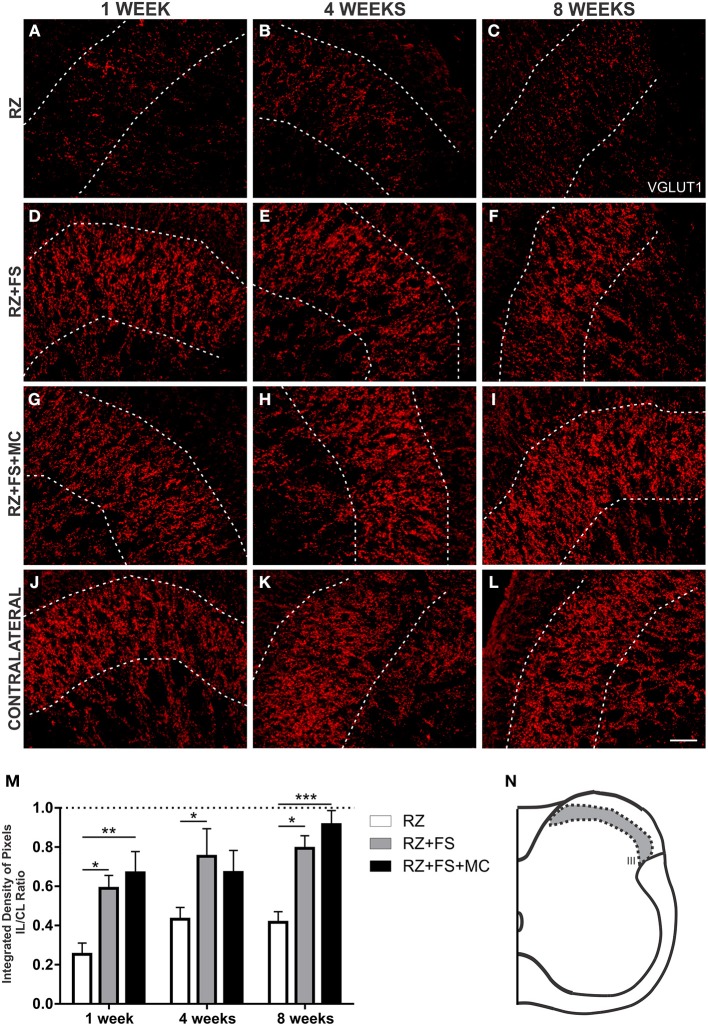
**Immunohistochemistry for vesicular glutamate transporter 1 (VGLUT1). (A–L)** Representative images of RZ, RZ+FS and RZ+FS+MC, 1, 4, and 8 weeks post lesion. **(M)** Quantification (ratio IL/CL) of the integrated density of pixels, obtained in the region delimited by dashed lines. **(N)** Representation of lamina III (total quantification area 3.9 × 10^5^ μm^2^). CL, contralateral; FS, fibrin sealant; IL, ispsilateral; RZ, rhizotomy; MC, mononuclear cells. Scale bar = 50 μm. ^*^p < 0.05; ^**^p < 0.01; ^***^p < 0.001.

Since primary afferents are VGLUT1 positive, we have used such marker to analyze the synaptic plasticity of such axons following root repair. The quantification in lamina III showed a decreased expression in RZ group, confirming the surgical procedure. There was an increased punctate labeling both in RZ+FS and RZ+FS+MC groups in comparison to RZ alone, 1, 4, and 8 weeks post-surgery (Figure 8). The mean ± SE of the groups are shown in Table S6.

**Figure 9:**
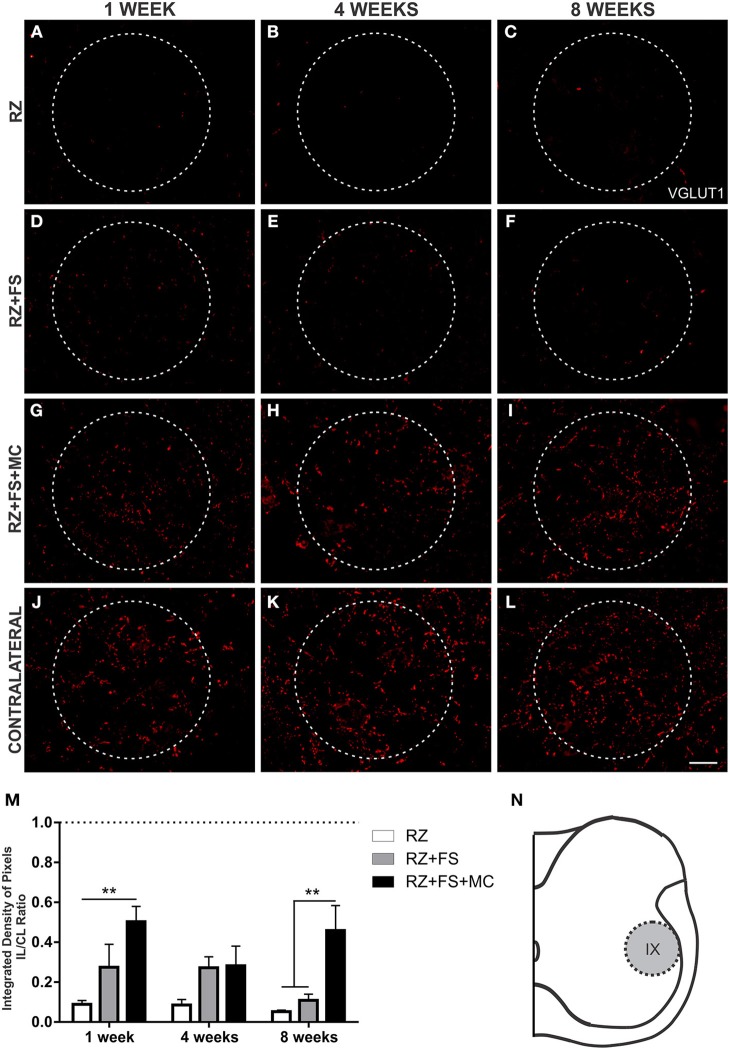
**Immunohistochemistry for vesicular glutamate transporter 1 (VGLUT1). (A–L)** Representative images of RZ, RZ+FS and RZ+FS+MC, 1, 4, and 8 weeks post lesion. **(M)** Quantification (ratio IL/CL) of the integrated density of pixels, obtained in the region delimited by dashed circles. **(N)** Representation of lamina IX (total quantification area 8.5 × 10^5^ μm^2^). CL, contralateral; FS, fibrin sealant; IL, ispsilateral; MC, mononuclear cells; RZ, rhizotomy. Scale bar = 50 μm. ^**^p < 0.01.

There was a reduction in VGLUT1 labeling at lamina IX in RZ group (Figure 9). An important finding of the present work was the significant presence of VGLUT1 positive nerve terminals in the surroundings of motoneurons at lamina IX, following MC therapy, 1 and 8 weeks post implantation (*p* < 0.01) (Figure 9). The mean ± SE of the groups are shown in Table S7.

**Figure 10:**
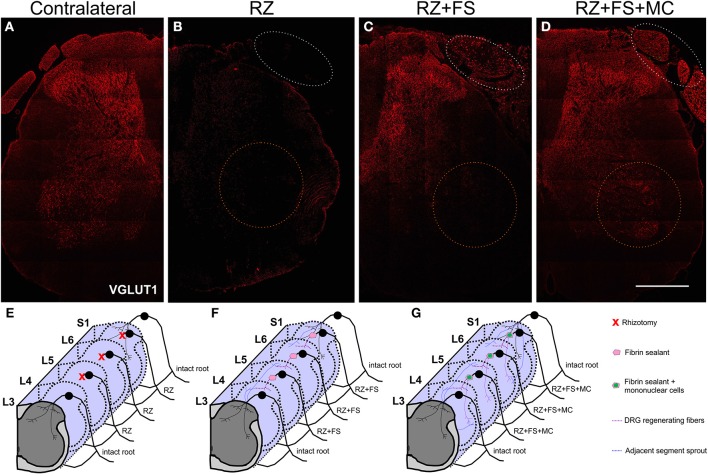
**(A–D)** Immunohistochemistry for VGLUT1. **(A)** Contralateral side. **(B)** IL side of RZ alone group. **(C)** IL side of RZ+FS group. **(D)** IL side of RZ+FS+MC group. White dotted ellipse represents the injury/reimplantation site. Orange dotted circle represents lamina IX. **(E–G)** Schematic representation of the proposed regeneration process following dorsal root reimplantation with fibrin sealant associated with mononuclear MC therapy. **(E)** RZ group. **(F)** RZ+FS group. **(G)** RZ+FS+SC group. FS, fibrin sealant; IL, ispsilateral; MC, mononuclear cells; RZ, rhizotomy. Scale bar = 500 μm.

**Figure 11:**
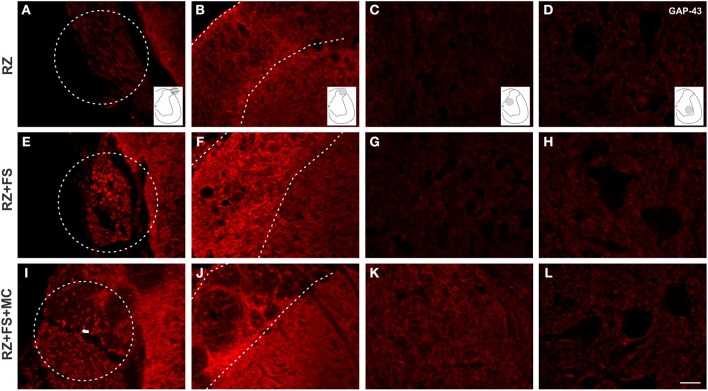
**Immunohistochemistry for GAP-43. (A–L)** Representative images of RZ, RZ+FS and RZ+FS+MC, 4 weeks post lesion. The location at the dorsal root area (dashed circles) and within the spinal cord (dashed lines) is indicated by the schematic drawings. Observe the increased number of positive axons toward deeper laminae following reimplantation of dorsal roots associated with cell therapy. FS, fibrin sealant; IL, ispsilateral; MC, mononuclear cells; RZ, rhizotomy. Scale bar = 50 μm.

Figure 10 shows panoramic transverse sections of the spinal cord in the different experimental groups, 8 weeks post lesion and repair. It is possible to observe a more intense immunofluorescence against VGLUT1 in the RZ+FS and RZ+FS+MC groups at the lesion site (Figures 10C,D, white dotted ellipses). It also important to point out the presence of MC on Figure 10D (in green), confirming the lesion site. Only in the RZ+FS+MC group, the immunostaining of afferents extended up to lamina IX (Figures 10C,D, orange dotted circles). Such results were further confirmed with GAP-43 immunolabeling. In this sense, growing axons could be seen in greater density in the groups RZ+FS and RZ+FS+MC (Figure 11). Such positive fibers were more numerous in deeper laminae only in RZ+FS+MC group.

A summary of the proposed synaptic plasticity within the spinal cord is presented in Figures 10E–G.

### GABAergic synaptic changes after lesion and root reimplantation

**Figure 12:**
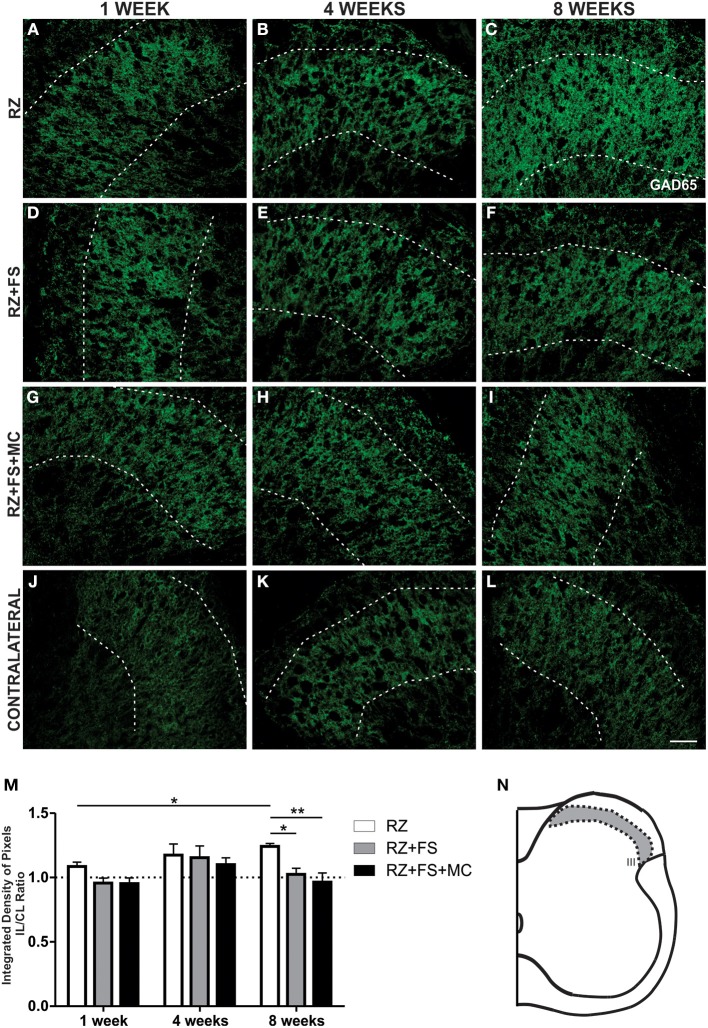
**Immunohistochemistry for GAD65. (A–L)** Representative images of RZ, RZ+FS and RZ+FS+MC, 1, 4, and 8 weeks post lesion. **(M)** Quantification (ratio IL/CL) of the integrated density of pixels, obtained in the region delimited by dashed lines. **(N)** Representation of lamina III (total quantification area 3.9 × 10^5^ μm^2^). CL, contralateral, FS, fibrin sealant; IL, ispsilateral; MC, mononuclear cells; RZ, rhizotomy. Scale bar = 50 μm. ^*^p < 0.05; ^**^p < 0.01.

Figure 12 shows the immunohistochemistry for GAD65, a GABAergic synapse marker. An increased density of GAD65 positive synapses in lamina III was observed in the RZ group, 8 weeks post lesion, compared to the same group 1 week following root cut (*p* < 0.05). Also, RZ group, 8 weeks post lesion, presented a significantly greater labeling in comparison to RZ+FS and RZ+FS+MC (*p* < 0.05 and 0.01, respectively). The mean ± SE of the groups is shown in Table S8.

No significant overall changes in the spinal cord circuit density were observed following synaptophysin labeling (Figure S2 and Tables S9–S11).

## Discussion

Avulsion of dorsal roots following brachial plexus injuries leads to paresthesia of the ipsilateral upper limb as well as results in loss of proprioception. In some instances, such lesions of the primary afferents produce neuropathic pain, which is usually difficult to be treated. Synaptic changes in the spinal cord network are still poorly understood (Darian-Smith et al., 2010). Regeneration of the dorsal root ganglia may enhance the untreatable pain since it may give rise to aberrant sprouting (Carlstedt, 2009) mostly at superficial laminae within the spinal cord. The present work addressed this issue by combining the reimplantation of dorsal roots with mononuclear cells and the results indicate that such approach improves the immunoreactivity against different synapse markers, increasing the labeling at deeper laminae within the spinal cord.

Different therapies have been proposed to reconnect spinal roots, such as autografts (Dam-Hieu et al., 2002) and the use of enzymes able to degrade extracellular matrix components (Sykova et al., 2006). Also, the use of Schwann cells, neural and embryonic stem cells has been suggested. However, such therapies are not feasible due to technical issues, such as difficulties in finding donor nerves with the similar caliber of the lesioned root, and the possibility to generate aberrant sprouting (Carlstedt, 2009). Also, the use of Schwann cells is impractical due to the difficulties in culturing such cells (Goel et al., 2009). The use of embryonic stem cells raises ethical problems and neural stem cells are difficult to extract because they are deeply located into the brain (Goel et al., 2009). Moreover, cell therapy has been used to treat spinal cord injury, but not dorsal root lesions (Sykova et al., 2006; Lu et al., 2012).

The fibrin sealant used in this work was selected because of its capacity of promoting homeostasis, preventing fluid loss and facilitating tissue adhesion (Barros et al., 2009). Such characteristics may have facilitated the growth of regenerating axons originated from the lesioned root, as indicated by Figure 10 (white dotted circles). Mononuclear cells were used based on the neuroprotective role in peripheral nerve regeneration (Goel et al., 2009) and because the ability to modulate inflamatory response by releasing anti-inflammatory cytokines (Ozdemir et al., 2012). Mononuclear cells are also easy to obtain, with no need of culturing (Goel et al., 2009). Thus, the association of a fibrin scaffold produced by the sealant, together with MC is proposed as an efficient strategy to reimplant lesioned dorsal roots.

In order to evaluate the overall synaptic immunoreactivity in the spinal cord we have used synaptophysin antiserum. Overall, no changes could be observed in the dorsal (laminae I and II, V, and VI medial part) and ventral horn (lamina IX) ipsilateral to injury. These results indicate that the global immunoreactivity against such synaptic marker is apparently unchanged following rhizotomy. In this way, the loss of excitatory inputs may be compensated by synaptic rearrangements or increased projections not analyzed in this work.

It is well known that dorsal root ganglion (DRG) neurons are glutamatergic and express the vesicular glutamate transporter type 1 (VGLUT1) (Li et al., 2003; Oliveira et al., 2003; Alvarez et al., 2010). In the spinal cord, on the other hand, most of the glutamatergic neurons are VGLUT 2 positive (Oliveira et al., 2003). In this sense, a dorsal rhizotomy is able to abolish almost all of the VGLUT1 positive synapses in the spinal cord, from the superficial layers down to the ventral horn, as shown in RZ samples, what it is also in accordance with previous work (Oliveira et al., 2003). Rats treated with FS and MC presented VGLUT1 labeling, what suggests synaptic plasticity within the affected spinal cord segment. Most of the recovered VGLUT 1 positive fibers may be derived from sprouting of fibers from other non-injuried segments. Interestingly, a recent work (Du Beau et al., 2012) has shown that there are VGLUT1 positive efferents from the corticospinal tract and other efferent pathways. Such descending fibers are not transected following dorsal rhizotomy and could be a source of sprouts that may account for part of the VGLUT1 positive terminals recovery found herein, following root reimplantation. Nevertheless, we believe that VGLUT 1 positive synapses may also originate from the DRG regenerating fibers, since the distance between the spinal cord adjacent segments is significantly greater than the reimplanted roots. This is also supported by the observation of afferent regenerating fibers in both reimplanted groups at the lesion site (Figures 10, 11). However, the use of anterograde tracing to label directly the afferent sprouts would give further proof of such reinnervation. However, since L4–L6 dorsal root ganglia are at least three vertebrae caudal to the lumbar intumescence, their dissection for tracer injecting, combined with dorsal root reimplantation would need large exposition of the spinal cord. This was not done due to ethical issues since the spinal cord could collapse after surgery, because of lack of bone support. Nevertheless, GAP-43 immunolabeling showed regrowing axons within the reimplanted roots, as well as in superficial and deep laminae following cell therapy. Such labeling was more intense following mononuclear cell therapy, particularly in deeper layers of the spinal cord.

The γ-aminobutric acid (GABA) is the major inhibitory neurotransmitter in the superficial regions of the spinal cord. The main functions of GABAergic neurons are presynaptic inhibition of primary afferents and postsynaptic inhibition of dorsal horn interneurons, motoneurons and sensory projections in the ventral horn (Darian-Smith et al., 2010). Inhibitory synapses were assessed by GAD65 immunostaining and revealed a compensatory increase following rhizotomy that is in line with the literature (Darian-Smith et al., 2010). However, it is still uncertain what triggered this effect, but it could be interpreted as a way to minimize the spinal cord circuits remodeling after an injury, because it is known that such plasticity leads to chronic pain. Thus, lowering the chances of generating aberrant sprouting may prevent neuropathic pain. The increase in GABAergic staining could also be explained by neurogenesis of inhibitory interneurons that was suggested following DRG lesions (Vessal et al., 2007), although it was not assessed in our work.

Importantly, the reimplantation of the dorsal roots leaded to a significant decrease in GFAP and Iba1 labeling, suggesting that this procedure minimizes reactive gliosis, which may in turn contribute to less formation of scar tissue resulting in a more permissive environment for synapse formation and stability. The reduction of gliosis may allow that neurotrophic cues, produced by interneurons and motoneurons in the spinal cord, can be available for the growing axons in the spinal cord microenvironment. It is known that motoneurons produce neurotrophic factors such as Neurotrophin-3 (Alvarez et al., 2010) that is required for sensory inputs stabilization (Mendell et al., 1999). Similarly, mononuclear cells are a source of neurotrophic factors that may direct the axons to the target (Goel et al., 2009), although this was not assessed in this work. Additionally, it was not observed any evidence that MC differentiated into another cell type, as shown in another study (Moraleda et al., 2006). Also, similarly to other work (Cabanes et al., 2007), it is uncertain if such cells differentiated into other cell types, such as microglia or macrophages.

The development of therapies that not only assist synaptic and glial recovery, but also result in functional improvement, is of fundamental importance. For this purpose, we have chosen to evaluate the recovery of nociceptive sensory function using electronic von-Frey test. Such test evaluates the mechanical nociceptive threshold, so that stimulation of mechanoreceptors occurs primarily by the pressure, but as the stimulus increases, the activation of C fibers and Aδ pain fibers also occurs. Thus, it was possible to mechanically stimulate the dorsal root dermatomes and to evaluate sensory response up to 1 month following reimplantation. Animals from the RZ+FS+MC group presented the best performance in such test. That was concomitant with the better reorganization of VGLUT1 fibers into deeper laminae of the spinal cord. It is known that the sensory inputs, when contacting directly motoneurons, are important for the arc reflex to work properly. Such synapses found around motoneurons might come from sensory neurons, VGLUT 1 positive, that have recovered post lesion.

To date, the Catwalk system® (walking track test, Noldus, The Nederlands) has only been used to evaluate motor recovery, such as in CNS lesion models (ventral root injury, lateral funiculus lesion, contusion, spinal cord transection) or in peripheral nerve injury (neurotmesis, nerve crush and transection) (Available in: http://www.noldus.com/CatWalk-XT/research-testimonials Access: 30 mar 2014) and allodinia (Vrinten and Hamers, 2003). However, there are no references showing that it may be useful to analyze lack of proprioception as seen herein. The results of the present study reinforce the importance of the sensory component for the correct motor coordination and motor control. The lack of sensory information triggered significant motor changes in RZ group. The motor dysfunctions seen in the present study can be primarily explained because of the loss of Ia afferent fibers. Such fibers are responsible for inhibiting the antagonist muscles during the gait process (Carlstedt, 2009).

Interestingly, despite that supraspinal afferents could not be reestablished solely by root reimplantation, spinal circuits recovered at a certain degree in treated groups. This is sufficient to improve motor function. Additionally, no behavioral indication of allodinia or hyperalgesia could be observed following reimplantation, what is in accordance with previous work confirming that dorsal root rhizotomy model does not trigger neuropathic pain (Sukhotinsky et al., 2004).

In conclusion, the dorsal root reimplantation with fibrin sealant, associated with bone marrow mononuclear cells decreased the glial reaction and prevented GABAergic inputs to over sprout, facilitating the recovery of VGLUT1 primary afferents (GAP-43 positive in reimplanted roots). This may in turn significantly improved motor and sensory function. Nevertheless, future studies are necessary to further understand, at cellular and molecular levels, the role of FS and MC. We believe that the present findings bring a new perspective on the possibility of repairing dorsal roots without generating neuropathic pain and aberrant sprouting, improving the motor coordination recovery.

## Author contributions

All authors had full access to all the data in the study and take responsibility for the integrity of the data and the accuracy of the data analysis. Study concept and design: Alexandre L. R. de Oliveira. Acquisition of data: Suzana U. Benitez, Aline B. Spejo and Roberta Barbizan. Analysis and interpretation of data: Suzana U. Benitez and Alexandre L. R. de Oliveira. Drafting of the manuscript: Suzana U. Benitez and Alexandre L. R. de Oliveira. Critical revision of the manuscript for important intellectual content: Suzana U. Benitez and Alexandre L. R. de Oliveira. Statistical analysis: Suzana U. Benitez. Obtained funding: Alexandre L. R. de Oliveira. Administrative, technical, and material support: Rui S. Ferreira Jr., Benedito Barraviera and Alexandre L. R. de Oliveira. Study supervision: Alexandre L. R. de Oliveira.

### Conflict of interest statement

The authors declare that the research was conducted in the absence of any commercial or financial relationships that could be construed as a potential conflict of interest.
