# Supplementary material for: Synaptic plasticity and sensory-motor improvement following fibrin sealant dorsal root reimplantation and mononuclear cell therapy
Source: Front Neuroanat. 2014 Sep 9;8:96. doi: 10.3389/fnana.2014.00096 (PMC4158877; doi:10.3389/fnana.2014.00096)
Supplement: Supplementary file 4 [file Presentation1.PDF]

Table S1. Antibodies and the respective rexed laminae analyzed.

Antibody	Analyzed laminae
Synaptophysin	I and II, V and VI, IX
VGLUT1	III, V and VI, IX
GAD65	III
GFAP	I and II, IX
GAP-43	Dorsal roots, laminae I and II, V and VI, IX

Table S2. GFAP immunolabeling quantification at laminae I and II (integrated density of pixels). The values are presented as mean ± standard error.

Survival time\Group	RZ/n	RZ+FS/n	RZ+FS+MC/n
**1 week**	2.19±1.19/5	2.15±0.57/5	2.45±0.33/6
**4 weeks**	4.66±1.22/5	2.63±0.32/5	1.83±0.19/5
**8 weeks**	5.81±1.38/5	3.38±0.65/6	2.43±0.25/5

FS, fibrin sealant; MC, mononuclear cells; RZ, rhizotomy.

Table S3. GFAP immunolabeling quantification at lamina IX (integrated density of pixels). The values are presented as mean ± standard error.

Survival time\Group	RZ/n	RZ+FS/n	RZ+FS+MC/n
**1 week**	1.84±0.25/5	1.62±0.31/5	1.73±0.26/5
**4 weeks**	1.66±0.11/5	2.27±0.61/5	1.49±0.08/5
**8 weeks**	3.36±0.55/5	1.80±0.25/6	1.40±0.15/5

FS, fibrin sealant; MC, mononuclear cells; RZ, rhizotomy.

Table S4. Iba1 immunolabeling quantification at laminae I and II (integrated density of pixels). The values are presented as mean ± standard error.

Survival time\Group	RZ/n	RZ+FS/n	RZ+FS+MC/n
**1 week**	4.34±0.55/5	2.94±0.33/5	4.04±0.31/5
**4 weeks**	2.57±0.15/6	1.79±0.17/5	1.75±0.18/5
**8 weeks**	3.24±0.50/6	2.30±0.26/6	1.56±0.15/5

FS, fibrin sealant; MC, mononuclear cells; RZ, rhizotomy.

Table S5. Iba1 immunolabeling quantification at lamina IX (integrated density of pixels). The values are presented as mean ± standard error.

Survival time\Group	RZ/n	RZ+FS/n	RZ+FS+MC/n
**1 week**	1.84±0.13/5	1.43±0.11/5	1.46±0.06/5
**4 weeks**	1.48±0.07/5	1.63±0.22/5	1.31±0.05/5
**8 weeks**	1.71±0.23/6	1.42±0.10/6	1.25±0.16/5

FS, fibrin sealant; MC, mononuclear cells; RZ, rhizotomy.

Table S6. VGLUT1 immunolabeling quantification at lamina III (integrated density of pixels). The values are presented as mean ± standard error.

Survival time\Group	RZ/n	RZ+FS/n	RZ+FS+MC/n
**1 week**	0.25±0.06/5	0.59±0.07/5	0.67±0.11/6
**4 weeks**	0.43±0.06/5	0.75±0.14/5	0.67±0.11/5
**8 weeks**	0.42±0.05/5	0.79±0.06/6	0.91±0.07/5

FS, fibrin sealant; MC, mononuclear cells; RZ, rhizotomy.

Table S7. VGLUT1 immunolabeling quantification at lamina IX (integrated density of pixels). The values are presented as mean ± standard error.

Survival time\Group	RZ/n	RZ+FS/n	RZ+FS+MC/n
**1 week**	0.09±0.02/5	0.27±0.12/5	0.50±0.08/5
**4 weeks**	0.09±0.03/5	0.27±0.06/5	0.28±0.10/5
**8 weeks**	0.05±0.01/5	0.11±0.03/6	0.46±0.13/5

FS, fibrin sealant; MC, mononuclear cells; RZ, rhizotomy.

Table S8. GAD65 immunolabeling quantification at lamina III (integrated density of pixels). The values are presented as mean ± standard error.

Survival time\Group	RZ/n	RZ+FS/n	RZ+FS+MC/n
**1 week**	1.09±0.03/5	0.96±0.04/5	0.95±0.04/5
**4 weeks**	1.17±0.09/5	1.15±0.09/5	1.10±0.05/5
**8 weeks**	1.24±0.02/6	1.02±0.05/5	0.96±0.07/5

FS, fibrin sealant; MC, mononuclear cells; RZ, rhizotomy.

Table S9. Synaptophysin Immunolabeling Quantification At Laminae I and II (Integrated Density of Pixels). The Values Are Presented As Mean ± Standard Error.

Survival Time\Group	RZ/n	RZ+FS/n	RZ+FS+MC/n
**1 week**	0.89±0.08/5	1.06±0.05/5	1.16±0.05/6
**4 weeks**	1.19±0.13/6	1.16±0.10/6	0.99±0.04/5
**8 weeks**	1.12±0.10/5	1.15±0.15/6	1.16±0.04/5

FS, fibrin sealant; RZ, rhizotomy; MC, mononuclear cells.

Table S10. Synaptophysin Immunolabeling Quantification At Laminae V and VI (Integrated Density of Pixels). The Values Are Presented As Mean ± Standard Error.

Survival time\Group	RZ/n	RZ+FS/n	RZ+FS+MC/n
**1 week**	1.11±0.06/4	1.13±0.12/5	0.97±0.07/5
**4 weeks**	0.95±0.08/5	1.07±0.05/6	1.00±0.06/5
**8 weeks**	0.88±0.11/6	0.97±0.10/6	0.93±0.06/5

FS, fibrin sealant; RZ, rhizotomy; MC, mononuclear cells.

Table S11. Synaptophysin Quantification At Lamina IX (Integrated Density of Pixels). The Values Are Presented As Mean ± Standard Error.

Survival time\Group	RZ/n	RZ+FS/n	RZ+FS+MC/n
**1 week**	0.85±0.04/5	0.94±0.08/5	0.92±0.05/5
**4 weeks**	0.84±0.04/6	0.90±0.08/6	0.86±0.11/5
**8 weeks**	0.75±0.08/5	0.90±0.08/5	0.83±0.05/5

FS, fibrin sealant; RZ, rhizotomy; MC, mononuclear cells.
