# Supplementary material for: Synaptic plasticity and sensory-motor improvement following fibrin sealant dorsal root reimplantation and mononuclear cell therapy
Source: Front Neuroanat. 2014 Sep 9;8:96. doi: 10.3389/fnana.2014.00096 (PMC4158877; doi:10.3389/fnana.2014.00096)

Fig. S1 Characterization of the bone marrow mononuclear cells by flow cytometry.A. Percentage of expression of CD11b marker. B. Percentage of expression of CD3 marker. C. Percentage of expression of CD45 marker. D. Percentage of expression of CD34 marker. Black curve = negative control. Red curve = positive cells.

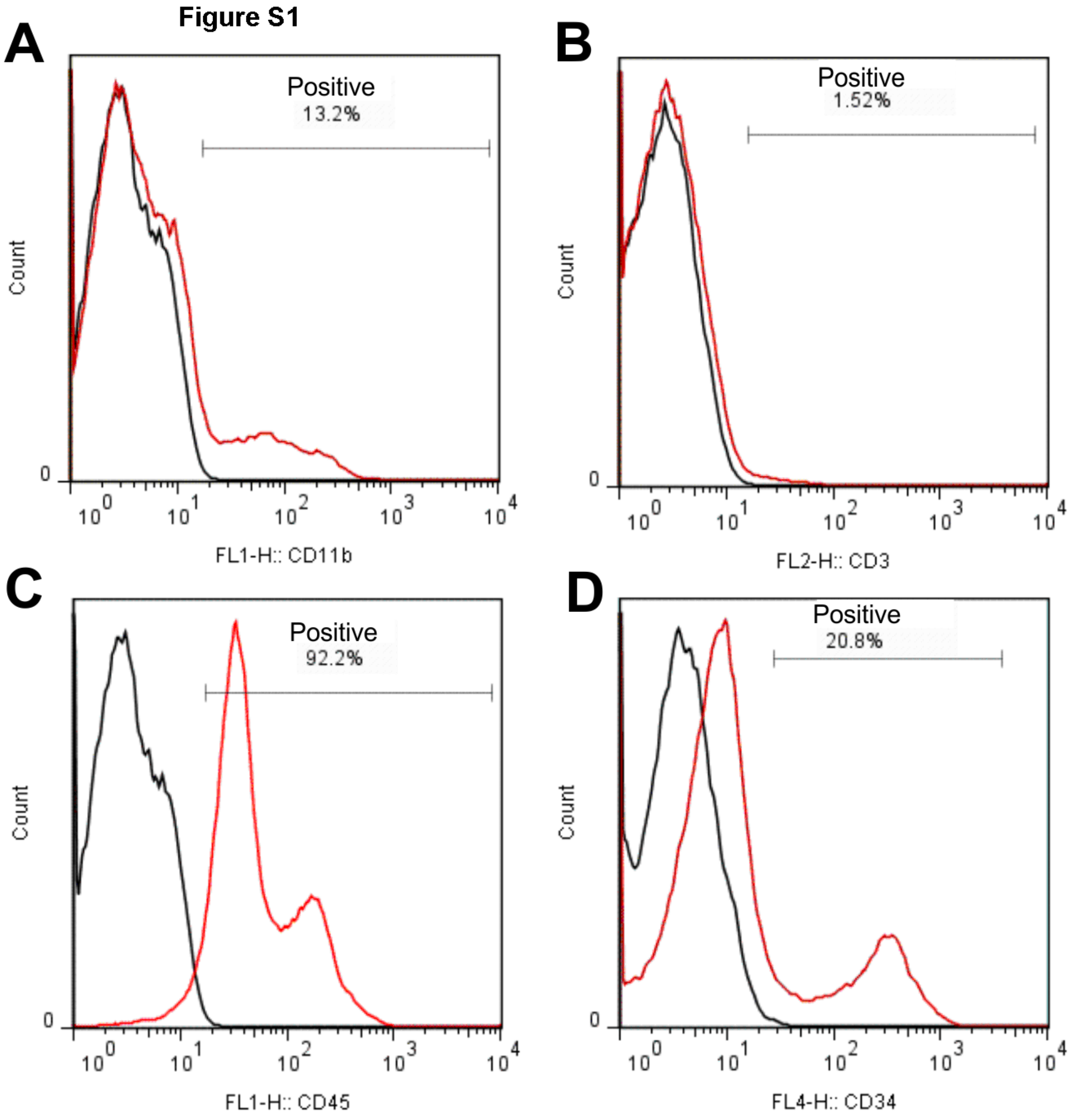

Supplement: Supplementary file 5 [file Image1.PDF]
