# Supplementary material for: Synaptic plasticity and sensory-motor improvement following fibrin sealant dorsal root reimplantation and mononuclear cell therapy
Source: Front Neuroanat. 2014 Sep 9;8:96. doi: 10.3389/fnana.2014.00096 (PMC4158877; doi:10.3389/fnana.2014.00096)

**Fig. S2** Immunohistochemistry for Synaptophysin. **A, D, G**. Immunolabeling at laminae I and II (dashed lines), V and VI (dashed circle), and IX, respectively (dashed circle). **B, E, H**. Representation of the analyzed Rexed laminae.**C, F, I**. Quantification (ratio IL/CL) of the integrated density of pixels. CL, contralateral, FS, fibrin sealant; IL, ispsilateral; RZ, rhizotomy; MC, mononuclear cells.Scale bar = 50 μm.

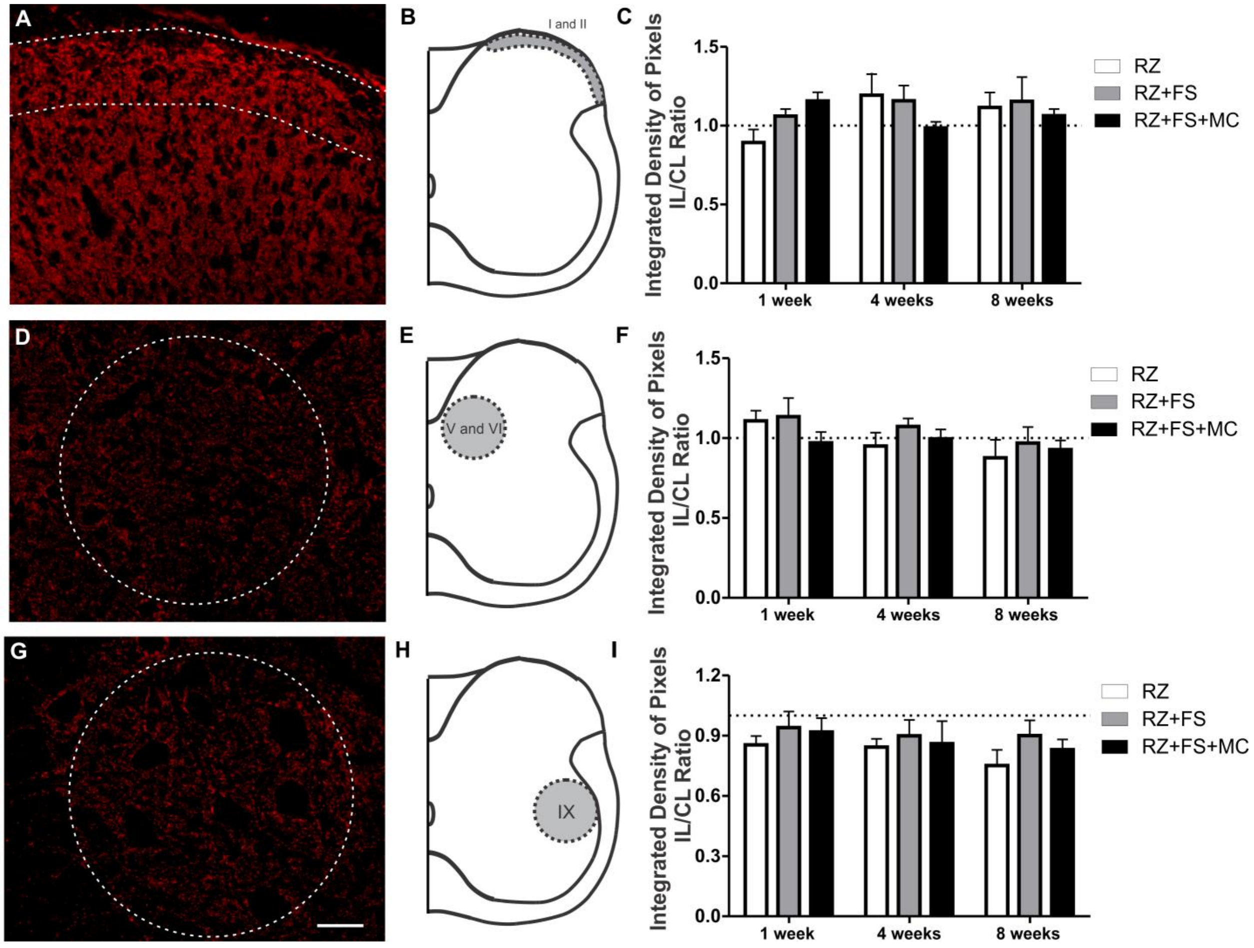

Supplement: Supplementary file 6 [file Image2.PDF]
